# Pathways to medical abortion self-use (MASU): results from a cross-sectional survey of women’s experiences in Kenya and Uganda

**DOI:** 10.1186/s12905-023-02570-2

**Published:** 2023-08-04

**Authors:** Ogol Japheth Ouma, Edward O. Ngoga, Isaac Odongo, Biko Steve Sigu, Angela Akol

**Affiliations:** 1 Ipas Africa Alliance, Research and Learning Advisor, P.O. Box 1192-00200, City Square, Nairobi, Kenya; 2 Ipas Africa Alliance, Quality of Care Manager, P.O. Box 1192-00200, City Square, Nairobi, Kenya; 3 Ipas Africa Alliance, Quality of Care Manager, Uganda, P.O. Box 1192-00200, City Square, Nairobi, Kenya; 4 Ipas Africa Alliance, Quality of Care Advisor-UCD, P.O. Box 1192-00200, City Square, Nairobi, Kenya; 5 Ipas Africa Alliance, P.O. Box 1192-00200, City Square, Nairobi, Kenya

**Keywords:** Information, Medical abortion, Pharmacies, post-MA contraceptive use, Medical abortion self-use, Kenya, Uganda

## Abstract

**Background:**

In Kenya and Uganda, unsafe abortions are a leading cause of maternal mortality. The new WHO policy guidelines on the safe termination of pregnancies up to 9 weeks lack information on women’s experiences with self-administered medical abortion (MA), impeding the development of interventions to increase MA use. This study aimed to comprehend women’s experiences with MA in Kenyan and Ugandan pharmacies.

**Methods:**

A cross-sectional mixed-methods survey utilized data from medical registers in 71 purposefully identified pharmacies and clinics dispensing MA drugs between September and October 2021. Forty women who were MA users participated in focus group discussions. The main outcome variables were: sources of MA information, costs of MA services, complications from MA, pain management, follow-up rates, and use of post-MA contraception. Quantitative data were analyzed using Stata 15, while qualitative thematic analysis was conducted using Dedoose qualitative analysis software.

**Results:**

73.6% of 2,366 women got an MA, both in Kenya (79%) and Uganda (21%). Most (59.1%) were walk-in clients. Kenya had significantly more women referred for MA (49.9%) than Uganda (10.1%) (p 0.05). Friends and family members were the main sources of MA information. The median cost of MA was USD 18 (IQR 10–60.5) in Kenya and USD 4.2 (IQR 2–12) in Uganda. Most MA clients received pain management (89.6%), were followed up (81%), and received post-MA contraception (97.6%). Qualitative results indicated a lack of medicines, high costs of MA, complications, stigma, and inadequate training of providers as barriers to MA use.

**Conclusions and recommendations:**

Communities are a valuable information resource for MA, but only if they have access to the right information. A relatively weak health referral system in Uganda highlights the importance of pharmacies and clinicians collaborating to support clients’ abortion needs and contraceptive use after medical abortion (MA). Low client follow-up rates show how important it is to make sure pharmacy technicians know how to give MA correctly. Finally, it is crucial to strengthen the supply chain for MA products in order to eliminate cost barriers to access.

## Background

Though the World Health Organization (WHO) has developed policy guidelines on safe abortions for pregnancies up to 9 weeks of gestation (63 days) [1], Medical abortion (MA) self-use is yet to be fully operationalized in developing countries. In Kenya, unintended pregnancies have contributed to an increase in the number of cases of unsafe abortion, with an estimated 465,000 induced abortions occurring each year, resulting in 2.6 deaths per 1,000 women and girls from unsafe abortion [2]. Similarly, unsafe abortion remains a leading cause of maternal death, with approximately 4–6 deaths attributed to unsafe abortion occurring every day, resulting in 336 deaths of women and girls per 100,000 in Uganda [3]. While women in Kenya and Uganda can procure MA drugs with prescriptions through pharmacies with a doctor’s prescription, there is no documentation in Kenya and Uganda on women’s experiences with MA self-use, thus making it difficult to plan care and interventions for women and girls who need these MA services.

Despite advancements in increasing safe abortion access and reducing abortion stigma, barriers to safe abortion still exist, with the need for a clinical visit being a key impediment. However, evidence shows that non-clinic abortion self-care can be highly effective, and outcomes compare favorably with in-clinic protocols [4]. The WHO acknowledges the global shortage of health workers and recognizes how self-care for sexual and reproductive health, including self-management of abortion, can expand access to essential healthcare, especially for vulnerable and underserved populations [5]. Ideal access to MA in both countries would be through pharmacies that accept doctor’s prescriptions. However, MA services can be accessed at clinics through trained physicians or pharmacists, but pharmacists are only permitted to dispense for pregnancies less than 9 weeks. Some physicians prefer that clients visit clinics for prescriptions before proceeding to pharmacists for dispensing, even if the gestational age is nine weeks or less; this is an impediment to access. In these contexts, self-managed abortion care refers to a situation in which women and girls with a pregnancy of nine weeks or less can access and self-administer MA drugs as prescribed by their provider outside the pharmacy or clinics.

Self-medication is enshrined in many countries’ treatment-seeking habits, including in Kenya and Uganda, where medicine sellers are often the first point of contact for girls and women who have health-related needs [6]. It is only when an illness persists or a complication arises that many people seek help in hospitals, clinics, or from a specialized health care provider.

Although some women and girls will undergo a medical abortion completely on their own, most will want someone they can get accurate information from or someone who can offer social and emotional support [7]. Many women and girls continue to seek abortions in the informal sector due to a lack of information, a lack of safe space, a lack of social support and accompaniment, and other reasons that are unknown [8].

The youth’s need for information on Sexually Reproductive Health Rights (SRHR), including contraception and medical abortion, has grown, and they are now accessing various digital platforms for information on unwanted pregnancy prevention. Ipas’s Big Ideas III (BI3) project in Kenya (2019–2021) was implemented in the context of the development and testing of prototypes for service packages that enhance self-care in Nairobi and Kajiado Counties. Results from the project indicated that the provision of accurate information and social and emotional support to women and girls may enhance self-care [9]. However, it is unknown if this is feasible in the pharmacy setup, which is unlikely to provide privacy, confidentiality, and social support and accompaniment. Given the high rate of unsafe abortions in Kenya and Uganda, the restrictive abortion laws, the emergence of self-care as a mainstream approach to health care, and the cultural practice of seeking healthcare services from medicine vendors, it is essential to understand women’s experiences with medical abortion use in Kenya and Uganda.

This cross-sectional survey on women’s experiences with medical abortion self-use at pharmacies and clinics in Kenya and Uganda was conducted to explore Kenyan and Ugandan women’s experiences with medical abortion self-use. Specifically, the survey sought to (1) identify the sources of MA information, (2) determine the costs of MA services, (3) establish the complications, pain management, and follow-up rates, and (4) determine the uptake of post-MA contraception. This study specifically focuses on pharmacies, and this was a program-based decision, as only pharmacists were supported programmatically. Nonetheless, this study revealed additional information source pathways.

## Methods

### Study location

The survey was conducted in Kenya (Kisumu, Busia, Siaya, Vihiga, and Trans Nzoia Counties) and Uganda (Kawempe and Rubaga Divisions of Kampala) between September 2021 and October 2021. These five locations were selected based on Ipas’s ongoing support of public health facilities, particularly because they have doctors and nurses trained in the provision of comprehensive abortion care, including post-abortion contraception.

### Study Design and data sources

The cross-sectional survey used a mixed-methods design that included both qualitative and quantitative methods. Trained Ipas staff collected quantitative data from client medical record registers at pharmacies and private clinics selling MA pills to women of reproductive age (WRA) and providing post-abortion care (PAC) services. Focus Group Discussions (FGDs) with MA users were conducted only in Kenya in a qualitative approach to identify and validate the pathways that women and adolescent girls consider for both MA and post-MA contraceptive use. FGDs were chosen programmatically in Kenya due to its higher client caseload than Uganda, as well as the scope of the program evaluation, which was not a research-based study.

Ipas’s Nimechanuka website is one of the sources of medical abortion information and contraception [10]. Nimechanuka is a semi-autonomous digital platform within the Ipas Africa Alliance, run by youth for youth. The platform was first established in 2013 as a digital platform providing SRH information and referrals to young people who are social media users and has now grown into a youth platform that seeks to create networks and empower youth-led organizations in SRHR programming.

At the facility, no women or girls were surveyed. However, because they had consented to services, their data was reviewed anonymously, and their names and details were removed from the records before review by trained Ipas staff.

### Study participants, sampling, and sample size

Given the subject’s sensitivity, a convenience-based purposive sampling method was chosen. The survey specifically targeted registered pharmacists who were identified during consultative meetings with the boards of the Kenya and Uganda pharmacy associations. Private clinics with a pharmacy wing were included, as were pharmacies that sold at least 10 medical abortion products per month. As a result, we identified a sample of 56 pharmacies (five in Uganda and 51 in Kenya) and 15 private clinics (ten in Uganda and five in Kenya) that consented to participating in the study. In the country, all pharmacies and private clinics were legally registered. In addition, 40 MA users were identified through the pharmacy where they purchased MA drugs and invited to participate in FGDs. Pharmacists mobilized some MA users retrospectively via telephone to explain the research and Ipas’ intention to conduct FGDs with those who agreed to participate. This was a programmatic decision based on the fact that, unlike in Uganda, the majority of these women and girls lived in Kenya, where the majority of clients were located. Women and girls were eligible to participate in the FGD if they had independently purchased, used, or inquired about MA drugs or contraception use at pharmacies and private clinics and could provide informed consent.

### Data collection

Data from client medical registers at pharmacies and clinics was documented using a data abstraction tool by trained Ipas staff between September and October 2021. This instrument was designed by program staff, who then validated it prior to piloting and data collection. The tool mainly captured the sources of MA information, costs of MA services, complications from MA, pain management, follow-up rates, and use of post-MA contraception. On the other hand, the FGDs were used to document the pathways and decision-making processes that women and girls use to access medication abortion services. Four focus group discussions (FGDs) were held in Swahili. FGDs with MA users were held in quiet environments in Swahili to capture emerging experiences and pathways. All interviews were digitally recorded and manually transcribed. The focus groups were led by Ipas project staff in Kenya who had been trained in qualitative methodology using the program training manual. This is also why no FGDs were held in Uganda, where there was no expertise.

### Outcome measures

The Client Medical Registers were examined for the following outcome variables: sources of MA information, costs of MA services, complications from MA, pain management, follow-up rates, and use of post-MA contraception. The proportion of MA users who used a contraceptive method by country, the proportion of MA users by source of information, the median and range costs of MA by country, the proportion of MA users who experienced complications, received follow-up, and received pain management by country, and the percent determined the outcomes.

### Data analysis and management

Tables and graphs were used to present descriptive analyses for both dependent and independent variables. A chi-square analysis was used to determine whether there is a statistically significant difference in the proportion of clients referred and walk-ins between Kenya and Uganda. Data cleaning, management, and analysis were carried out using STATA, Version 15. To achieve the desired classification, variables were recoded. Independent researchers translated transcripts of qualitative information from FGDs to English for language equivalence before coding and analysis. Inductive and deductive coding was done independently by a minimum of two members of the evaluation team using Dedoose qualitative analysis software. We used a sample of 10% of the transcripts to develop the codebook and ensure the reliability of code applications between coders using inter-coder reliability testing in Dedoose. After finalizing the codebook, all transcripts were coded again by at least one member of the evaluation team. Following this, coded transcripts were exported to an Excel matrix, analyzed for emerging themes, and triangulated across data sources. We also examined the trends in the data across subgroups of the sample, such as age group and marital status.

### Ethics and consent

The Jaramogi Oginga Odinga Teaching and Referral Hospital (JOOTRH) Ethics and Scientific Research Committee reviewed and approved the study protocol, with approval number IERC/JOOTRH/464/21. Additional permits to conduct the study were obtained from the proprietors of the registered pharmacies, as well as individual signed informed consent from each FGD participant. Informed Consent was obtained from all the participants in the study. During the informed consent process, data collectors emphasized to participants that the study is voluntary and ensured that they understood the risks and benefits of the study and were ready and willing to participate. Client Medical Records were reviewed by pharmacists and private providers who consented to participate in the study. We hereby confirm that all methods were carried out in accordance with relevant guidelines and regulations and within the parameters of the approved protocol.

## Results

### Distribution of pharmacies and clinics

In total, 56 pharmacies and 15 private clinics were identified in both Kenya and Uganda. Kenya had the highest number of facilities (n = 56, 78.9%), of which 76.1% were pharmacies. On the other hand, Uganda had twice as many private clinics as pharmacies (see Table 1).

**Table 1:** Distribution of pharmacies and private clinics (n = 71)

Country	Pharmacies	Clinics	Total
	n (%)	n (%)	
Kenya	51 (76.1)	5 (7.5)	56 (78.9)
Uganda	5 (7.5)	10 (14.9)	15(21.1)
**Total**	**56 (83.6)**	**15 (22.4)**	**71 (100)**
**County/Division**			
Kisumu	12 (17.9)	3 (4.4)	15(21.1)
Trans Nzoia	11 (16.4)	1 (1.5)	12 (16.9)
Vihiga	11 (16.4)	1 (1.5)	12 (16.9)
Busia	10 (14.9)	-	10 (14.1)
Siaya	7 (10.4)	-	7 (9.8)
Rubaga	3 (4.6)	6 (9.0)	9 (12.7)
Kawempe	2 (3.0)	4 (6.0)	6 (8.5)
**Total**	**56 (83.6)**	**15 (22.4)**	**71 (100)**

### MA access and client type

A total of 2,366 women and girls visited the 71 pharmacies and clinics in Kenya and Uganda to purchase MA pills for abortion services during the survey period. Of these, 1,742 (73.6%) received MA pills (Table 2).

Most of the MA clients (n = 1,029; 59.1%) were walk-ins, i.e., had no referral from a medical practitioner. Kenyan women and girls were more likely than Ugandan women and girls to be referred for MA services; the proportion of Ugandan clients who were referred for MA pills in Uganda (10.1%) was significantly lower than the proportion of referred clients in Kenya (49.9%) (p = 0.005). As a participant from Kenya said, *“…. These pharmacists you can’t just go and visit them like that (direct walk-ins) and share your problems, you must find someone to refer you and make the initial connections for you to be served better*.“ *(MA User, 21 years, Kisumu County)*.

**Table 2:** MA Access, referrals, and walk-ins (n = 1,742)

Country	Number of MA clients	Number of referred clients	Number of walk-in clients
Kenya	1,377 (79.0)	676 (49.9)	701 (50.1)
Uganda	365 (21.0)	37 (10.1)	328 (89.9)
**Total**	**1,742**	**713**	**1,029**
**County/Division**			
Kisumu	377 (21.6)	171 (12.4)	206 (29.4)
Trans Nzoia	86 (4.9)	23 (1.7)	62 (8.8)
Vihiga	337 (19.4)	188 (13.7)	149 (21.5)
Busia	387 (22.2)	187 (13.6)	200 (28.5)
Siaya	190 (10.9)	107 (7.8)	84 (12.0)
Rubaga	171 (9.8)	0	171 (52.1)
Kawempe	194 (11.1)	37 (10.1)	157 (47.9)

### Sources of MA information

More than half (n = 744, 72.3%) of the walk-in MA clients learned about MA from friends, peers, and family members in both Kenya (n = 494, 70.5%) and Uganda (n = 250, 76.1%), while healthcare workers were the source of information for 15.8% of Kenyan and 21.7% of Ugandan clients (Table 3).

Results from the FGD support the findings. One of the participants in the FGD groups stated,

“……. *I work in a hair salon. We usually have a very interesting conversation about women’s health. We learn from personal experiences and challenges, including medical abortion.“ (MA User, 32 years, Vihiga County)*.

Consistently, some MA users learn about MA from family members, as observed during the FGDs, where one MA user had to say,

*“My husband keeps saying that the injection will make me disinterested in sex, so we were not using any family planning method but instead relied on safe days, which I may not be sure of, so one day after realizing that I got pregnant again, I shared with my husband, who asked whether it could be that I counted safe days wrongly! After exploring options, he shared with me about MA, which he also learned from other friends. (MA User, 30 years, Vihiga County)*

Similarly, another FGD participant noted,

“*That is when I went and shared with my mother that story, and my mother told me perhaps we should go and inquire from the chemist because I don’t want your father to know and I don’t want anyone to know that story; it is between me and you.” (MA User, 20 years, Busia County)*

While none of the pharmacists from Uganda reported Ipas’s Nimechanuka website as the source of MA information, 58 (8.3%) pharmacists from Kenya had data indicating that MA users learned about MA from the Nimechanuka Website.

In Kenya, 232 (34.3%) MA users learned about MA from youth champions. The corresponding proportion for Uganda is 75% (n = 28). Other MA users relied on previous clients, the internet, and other pharmacies for MA information (Table 3).

**Table 3:** Sources of MA information

Walk-in clients, (n = 1,029)					
Country	Friend/Peers	Healthcare Workers	Family Members	Nimechanuka Website	Others
Kenya	56.9	15.8	13.6	8.3	5.4
Uganda	57.0	21.7	19.1	-	2.2
**County/Division**					
Kisumu	61.3	3.2	22.6	9.7	3.2
Trans Nzoia	59.0	28.2	10.3	-	2.1
Vihiga	52.3	15.0	9.4	17.8	5.5
Busia	48.7	24.3	21.6	2.7	2.7
Siaya	68.6	9.8	13.7	3.9	4.0
Rubaga	74.4	1.1	24.4	-	0.1
Kawempe	48.9	31.2	16.7	-	3.2
**Referred clients, (n = 713)**					
**Country**	**Youth Champions**	**Other hospitals**	**Other pharmacies**	**Previous clients**	**Others**
Kenya	34.3	28.6	14.3	12.7	10.1
Uganda	75.0	16.6	8.3	-	0.1
**County/Division**					
Kisumu	-	-	33.3	33.4	33.3
Trans Nzoia	83.3	16.7	-	-	-
Vihiga	69.2	4.1	23.1	-	3.6
Busia	-	187 (100.0)	-	-	-
Siaya	-	-	33.3	33.3	33.4
Rubaga	-	-	-	-	-
Kawempe	57.1	14.3	28.6	-	-

### Costs of MA services

The median cost of a combi-pack in Kenya (USD 20.5; IQR 12.5–115) outweighs the median cost of a full dose of misoprostol (12 tabs) (USD 18; IQR 9–60). In Uganda, the median cost of a combi-pack is USD 6 (IQR 3–7.5), while the median cost of misoprostol is USD 2.6 (IQR 2–6). The cost of MA in Kisumu County, Kenya, is the highest, with a median of USD 62 (IQR 35–250) for a combi pack and USD 20 (IQR 15.2–24) for misoprostol. The cost of MA is lowest in Siaya County, with median costs of USD 7 (IQR 3.5–USD 10) for combi pack and USD 5 (IQR 4.8–USD 9.1) for misoprostol. In Uganda, the cost of MA is highest in the Kawempe division, with a median cost of USD 4.8 (IQR 4.5–USD 5.5) for the combi pack and USD 4 (IQR 1.8–4) for misoprostol.

The costs of MA were a concern for many girls and women. For instance, one of the FGD participants stated,

“*Reduce the cost of service to make it accessible to all women and girls seeking MA; this will reduce the number of people visiting the herbalists and quacks.“ (MA User, 28 years, Kisumu County)*.

The findings showed that some girls and women failed to secure contraception after MA use due to a lack of finances. For example, one of the FGD participants said,

*“I didn’t go for the Depo injection… I didn’t have money….“ (MA User, 21 years, Busia County)*.

In addition, another FGD participant said,

“*I paid almost USD 50, and I was given only 2 pills, of which I didn’t know their names or what to do with them. I tried to terminate but it didn’t work, so I decided to carry a pregnancy to term because I could not go back since it was expensive*.” *(MA User, 26 years, Busia County)*.

Additionally, it seems most pharmacists charge high prices for MA services, causing some MA users to negotiate costs. For instance, one of the FGD participants said,

“*My boyfriend directed me to a chemist; he had communicated with the provider and negotiated the prices. He got a place out of town; we were able to hide there until the process was complete*.” *(MA User, 26 years, Vihiga County)*.

### Pain management, follow-up, and complications

**Fig. 1:**
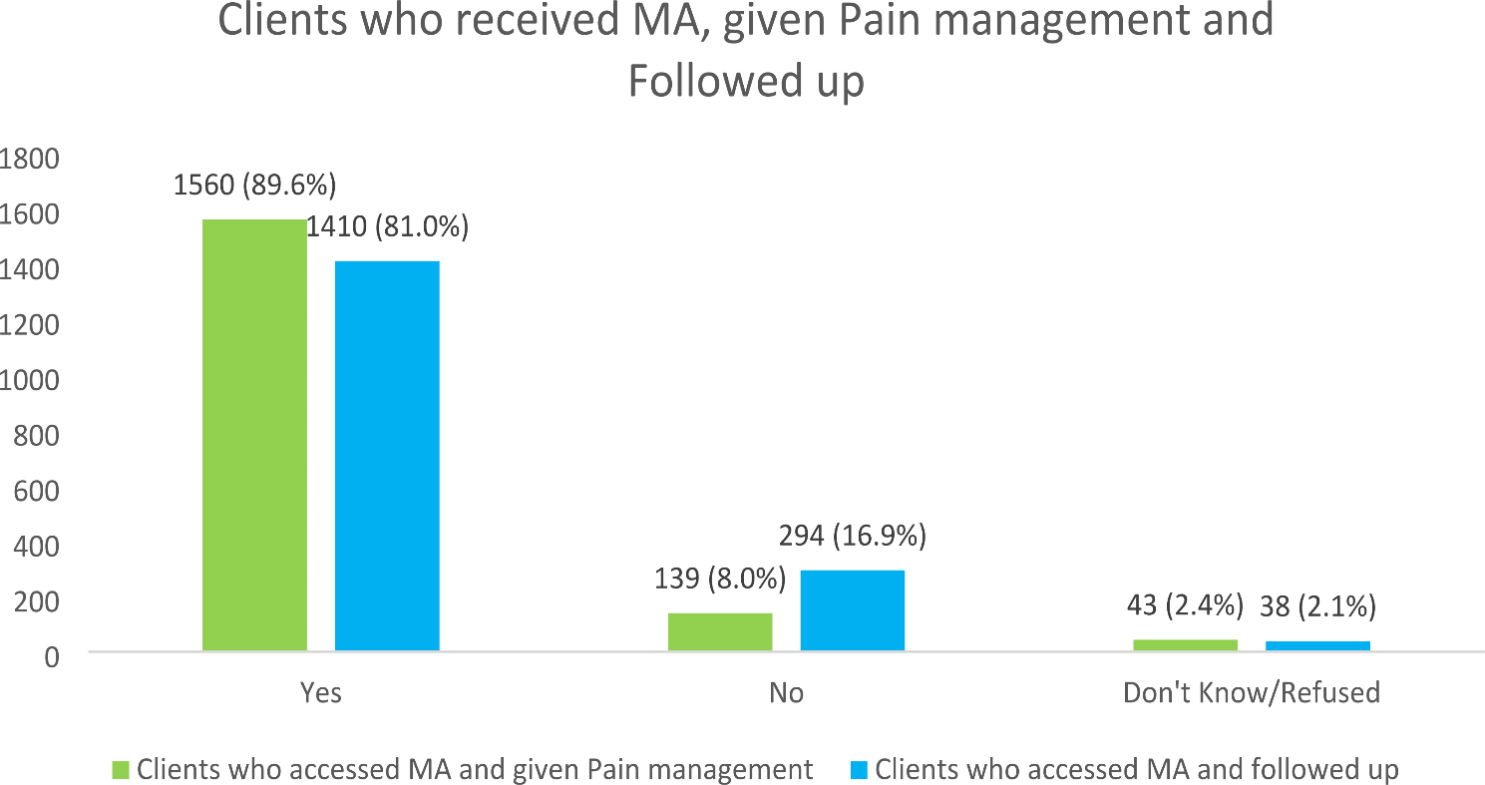
Pain management and clients follow up

Most MA clients (n = 1,560, 89.6%) received pain management (Kenya, 89.8%, and Uganda, 89.0%). In addition, the majority (n = 1,410, 81%) were followed up for complications (see Fig. 1). Uganda reported the lowest rate of client follow-up (54.5%).

Some pharmacists do not follow up with MA clients. For instance, one of the girls from FGDs quoted,

“*They never follow up, just dish out the drugs without proper guidance, and never want the clients to return to the facility.” (MA User, 23 years, Kisumu County)*.

FGD findings indicated that complications from MA use were a concern, as highlighted by participants.

*“Access to abortion pills over the counter at times leads to complications if not properly used, like underdose and poor instructions on how to use drugs after purchase.” (MA User, 25 years, Kisumu County)*.

“T*Train pharmacists to understand the procedure to reduce the risk of complications that may lead to disclosure to the community.“ (MA User, 27 years, Busia County)*.

The findings also revealed that pharmacists were unconcerned about gestational age as long as the user covered the costs.

“*Some pharmacists will still sell medical abortion pills to women who are 5 or 6 months pregnant, provided you meet the cost.” (MA User, 23 years, Vihiga County)*.

### Use of post-MA contraception

**Fig. 2:**
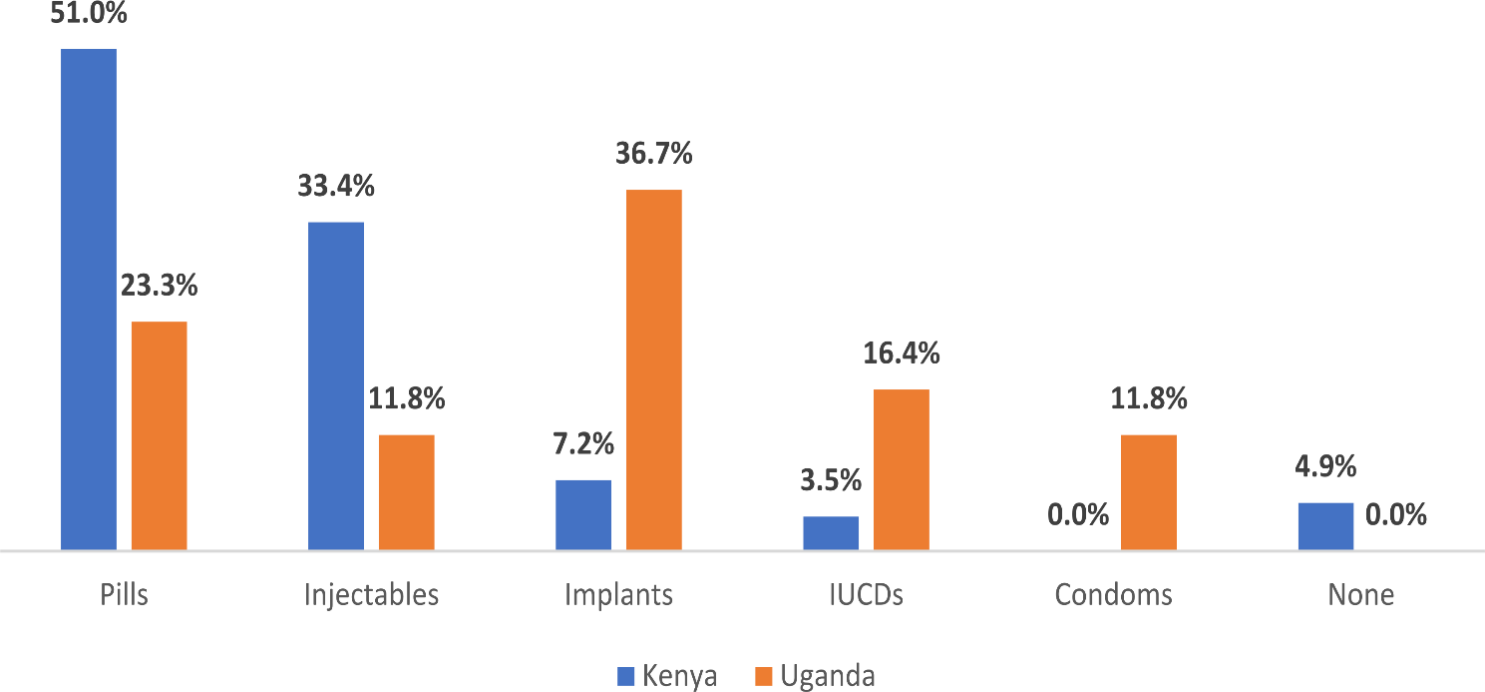
Post-medical abortion contraception by Country

Overall, 97.6% of MA clients left with a contraceptive method. All the MA clients in Uganda obtained contraceptives, compared to 95.1% of MA clients in Kenya. The most popular method was pills (n = 787, 45.2%), followed by injectables (n = 502, 28.8%). All 85 MA clients (4.9%) who did not receive any form of contraception were from Kenya (Fig. 2). Misconceptions about the efficacy of contraceptives were cited as a reason for not using contraception after MA use. For example, one of the FGD participants said,

“*My friends insist that contraceptives nowadays are fake, there is a girl in my community who had an implant but was able to conceive*.” *(MA User, 26 years, Vihiga County)*.

Also, another FGD participant quoted,

*“I can’t say I trust the contraception methods 100%, but I am willing to give them a try. The pharmacist counseled me and my husband on our options.“ (MA User, 32 years, Vihiga County)*.

## Discussion

The study sought to determine women’s pathways and experiences with MA use in Uganda and Kenya. We found that friends, peers, and family members are a prevalent source of information on MA, that most clients access MA without a referral, and that while pain management is nearly ubiquitous, client follow-up for complications management is poor. Furthermore, the cost variability of MA products constitutes a barrier to MA access.

Our study found that friends, peers, and family members are most relied on for MA information. The over-reliance on friends and peers for information may be due to the sensitivity of abortion care, leading women to trust friends and peers, especially when such friends have had experiences with abortion. This finding is consistent with the literature that shows that friends, peers, and family members are among the leading sources of health information, especially for MA users in Africa [7]. Similarly, in India, Agwuna and others (2018) found that the effective provision of health information by friends, peers, and family members from community information centers is critical for women and girls in need of medical abortion services [11]. The implications of this finding for empowering social networks with correct and accurate information find support in Rossier and colleagues (2021), who found that improved social networks enhanced access to medical abortion service provision in Kenya and beyond [8].

We found that most clients were walk-ins and that MA clients in Uganda were significantly less likely to have a referral for MA than Kenyan clients. This result correlates with the findings of Byansi et al., who found that most clients seeking healthcare services in Uganda are direct walk-ins [12]. As with the study of Byansi et al., medical abortion is a stigmatized service that is not widely available, which may affect the clients’ trajectory into care. The walk-ins may have been contributed by most clients learning about MA from friends and peers. This finding can be interpreted to imply a relatively strong referral system in Kenya and to represent a risk to the quality of MA care in Uganda.

On the other hand, we found that financial constraints, including the cost of accessing medical abortion services, are a barrier for most MA users. Similar studies from South Africa have found that women’s experiences with access to medical and surgical first-trimester abortion services are associated with costs as a barrier [13]. We also found that the costs of MA were highly variable between and within the two countries, potentially leading to delays in care or causing women to consider other alternatives for self-induction that may not be safe. The high cost of MA services at pharmacies, especially in Kenya, may be a contributor to the 20% of complications from unsafe abortion generally observed as women and girls resort to other unsafe methods for terminating pregnancies [14]. While standardizing the costs of medical abortion services may not be attainable given that pharmacies are business-oriented, linking pharmacies to certified MA suppliers may reduce wide variability when it comes to access, availability, and utilization of medical abortion services. This suggestion is confirmed by Footman et al. (2018), who recommend that pharmacists should be linked to medical abortion drug suppliers to reduce the costs of medical abortion services [14].

While our findings showed that almost 90% of MA users were given pain management in both Kenya and Uganda, the rate of client follow-up was low, especially in Uganda. In West Africa, follow-up is critical for MA clients, especially when it is conducted within one week since it helps understand the women’s experiences with medical abortion, including but not limited to any additional doses taken, warning signs of complications, completion of abortion, healthcare seeking, disclosure of the abortion, satisfaction with the accompaniment group, and emotions about the experience [15]. Therefore, a low follow-up rate may be a contributor to a high level of complications among women and girls who experience medical abortion in Uganda [16]. On the other hand, the finding is consistent with research showing that pain management is above 76% for patients who have undergone medical abortion compared to those who have been given surgical management for incomplete abortion in Uganda [17]. The opposite result was obtained in India and Nepal, where the likelihood of follow-up is high for most clients seeking abortion care through pharmacies [18].

Overall, our findings showed that 93.6% of MA clients left with a contraceptive method, with pills being the most popular method while LARC services such as IUDs and implants were the least popular. While the WHO recognizes that the provision of postabortion contraception is a promising strategy for reducing unintended pregnancy and associated maternal morbidity and mortality [19], the findings do not reflect the increasing use of LARC in developing countries compared to short-term methods [20]. The low uptake of LARC reflects the reality that most sites in this study were pharmacies that are not set up to provide LARC compared to clinics, which raises critical concerns for an informed choice of contraceptive methods obtained within pharmacies. Therefore, a strong referral network between pharmacists and clinicians and task-sharing should be enhanced to help refer women and girls in need of LARC services, as supported by the findings of Adedini and others in 2019 [19].

### Strengths and limitations

To our knowledge, this study is one of the very few attempts to explore women’s pathways and experiences with MA use in sub-Saharan Africa. A key strength of this study is the large sample size of users, considering the hidden nature of abortion in the two countries. Digitized data collection reduced data entry errors, especially when data collectors were required to type their own responses without skip logic, thereby improving data quality. Further, data was collected and entered in the medical registers by providers with whom clients have developed trust as part of the service delivery experience, increasing the likelihood of client responsiveness.

On the other hand, the use of clinic and pharmacy staff to collect information could potentially have introduced bias related to social acceptability. Further, the providers’ opinions while collecting the data could have influenced some responses. In addition, the four FGDs conducted were few and were based on available and willing participants. Given the nature of the topic, four was the most feasible number. Nevertheless, the themes obtained were uniform across.

## Conclusions

Pathways into MA use in Kenya and Uganda are characterized by a reliance on MA information from informal sources, cost barriers, and low rates of referral. The typical service experience is one of good pain management, poor client follow-up, and a strong reliance on post-MA contraception using short-term methods.

These findings suggest that communities are informal sources of critical health information for stigmatized services like abortion and need to be empowered with the right information on medical abortion. Findings also suggest a relatively weak health referral system in Uganda, highlighting the need for linkages between pharmacies and clinicians to address client abortion needs. Such a referral system would also support the use of LARC after MA. This finding also accentuates the need to enhance the capacity and knowledge of pharmacy technicians to dispense MA correctly and follow up with clients, given the preference for clients to walk into pharmacies for care. Lastly, strengthening the supply chain for MA products by linking pharmacists to suppliers and distributors of MA and contraceptive commodities is critically important to remove price variability and eliminate cost barriers to MA access.

## Data Availability

The dataset was collected through ODK and submitted to the Ipas Mobile Application Reporting System (MARS), where it was retrieved and cleaned for analysis. The data can be shared upon request to prof.japheth@gmail.com and cc akola@ipas.org given the sensitivity of the data.

## Abbreviations

FGDs: Focus Group Discussions
IUD: Intrauterine Device
IRB: Institutional Review Board
JOOTRH: Jaramogi Oginga Odinga Teaching and Referral Hospital
LARC: Long-Acting Reversible Contraceptives
MA: Medical Abortion
MASU: Medical Abortion Self-Use
PAC: Post Abortion Care
PMAC: Post Medical Abortion Contraception
USD: US Dollar
WHO: World Health Organization
WRA: Women of Reproductive Age
